# Editorial: Decoding immunologic complex systems in cancer: the cutting-edge technologies revolutionizing cancer immunology and immunotherapy

**DOI:** 10.3389/fimmu.2023.1208589

**Published:** 2023-05-04

**Authors:** Hu Li, Jin Zhang, Yuin Han Loh, Daniel D. Billadeau

**Affiliations:** ^1^ Department of Molecular Pharmacology and Experimental Therapeutics, Center for Individualized Medicine, Mayo Clinic, Rochester, MN, United States; ^2^ Center for Stem Cell and Regenerative Medicine, Department of Basic Medical Sciences and the First Affiliated Hospital, Zhejiang University School of Medicine, Hangzhou, China; ^3^ Institute of Hematology, Zhejiang University, Hangzhou, China; ^4^ Zhejiang Laboratory for Systems and Precision Medicine, Zhejiang University Medical Center, Hangzhou, China; ^5^ Cell Fate Engineering and Therapeutics Lab, Cell Biology and Therapies Division, ASTAR Institute of Molecular and Cell Biology, Singapore, Singapore; ^6^ Department of Biological Sciences, National University of Singapore, Singapore, Singapore; ^7^ Department of Physiology, Yong Loo Lin School of Medicine, National University of Singapore, Singapore, Singapore; ^8^ National University of Singapore (NUS) Graduate School for Integrative Sciences and Engineering, National University of Singapore, Singapore, Singapore; ^9^ Division of Oncology Research, College of Medicine, Mayo Clinic, Rochester, MN, United States; ^10^ Department of Immunology, College of Medicine, Mayo Clinic, Rochester, MN, United States

**Keywords:** cancer immunology, immunotherapies, systems cancer immunology, artificial intelligence, multi omics

Over the last decade we witnessed an arising number of technological innovations such as single-cell multi-omics technologies, combined with the democratization of artificial intelligence (AI) and development of sophisticated systems and network biology algorithms with their application in cancer immunological research (Meijerink et al.) (1), including immunotherapy (Dhillon et al.; Xie et al.). Despite these advancements, decoding the intricate nature of molecular and cellular interactions mediating immune cancer modulation and treatment responses remains a critical need in the field: indeed, our ability to leverage these cutting-edge technologies to unravel the complexity of the immune system, will permit us to harness learned lessons to better understand cancer etiology, and help design better immunotherapies.

This Research Topic aims at highlighting cutting-edge technologies that are set to revolutionize the field of immunology paving the way to systems cancer immunology. This includes rethinking and expanding research to better understand the complex immunologic landscape in cancer. For instance, how can knowledge gained from systems biology approaches, AI, and other disciplines including stem cell and synthetic biology be harnessed to enhance the development of immunotherapy options. Furthermore, the development of individualized medicine approaches to modulate the immune system in specific cancer patients will ultimately improve outcomes and treatment efficacy. The contributing articles in this Research Topic address these key questions.

Bridges and Miller-Jensen provide a comprehensive review on single-cell RNA-sequencing (scRNA-seq), highlighting methods developed to identify inter- and intracellular signaling from scRNA-seq, as well as strategies to integrate scRNA-seq-inferred interaction networks with existing experimental and computational approaches. This review offers an integrated view on how single-cell data and computational approaches bring novel insights to better understand immune response and immunotherapy-altered signaling in the tumor microenvironment (TME). More importantly, the authors identified advantages and some limitations of several major computational approaches used to infer cell-cell communication, including cellular spatial interactions in the TME. This resourceful review will facilitate single-cell research in cancer immunology and immunotherapy.

This follows by a review from Fan and Wu decoding lung cancer etiology at the single-cell level. The authors reviewed methods in single-cell analysis and the application in lung cancer research, to unveil the heterogeneity of lung cancer, at its role in shaping the TME which in turn dictates cancer invasion and metastasis. The authors also discussed the underlying mechanisms of drug resistance from a single-cell perspective. In sum, this review offers an overview and yet integrated perspectives on the applicability of single-cell analyses to advance our understanding in various cancerous phenotypic properties, including the promise of optimal personalized treatments in lung cancer and other human diseases.

Mi et al. employed an unbiased, integrative multi-scale spatial analysis to uncover how tumor and immune components interact in the TME and their role in determining the responsiveness of advanced hepatocellular carcinoma (HCC) to therapeutics. Their analysis framework comprises the quantification of intra-tumoral phenotypic heterogeneity, stratification of tissue architectures, multicellular protein expression analysis, and network analysis of cellular communities. Responder and non-responder cohorts to neoadjuvant cabozantinib and nivolumab treatment were analyzed. They found proximity of CD8^+^ T cells to arginase 1^hi^ (Arg1^hi^) macrophages, rather than CD4^+^ T cells within the TME of non-responders. This study also revealed distinct macrophage-enriched cellular communities and lymphocyte-enriched cellular communities in non-responders, indicating unique cellular communities in the TME contribute to shaping drug response phenotypes in HCC. Hence, results from this study can provide critical information for the development of mechanistic quantitative systems pharmacology models aimed at predicting clinical outcomes.

Zhang et al. developed a novel artificial neural network (ANN) based tool called artificial neural network encoder (ANNE) that can be used to extract knowledge, i.e., gene-gene associations that explain phenotypic behaviors, learned by ANN from big ‘omics’ data. The underlying algorithm, weight engineering, that is used to extract knowledge from trained ANN, is inspired from how the brain learns and stores information in the form of weighted neuronal connections among synapses. It is the “weights” associated with interneuronal connections that carry learned knowledge, and, by manipulating these weights, i.e., using weight engineering, we retrieve the information that is learned in the brain. The authors used a breast cancer dataset from patients with known clinical outcomes to test this idea on ANNE that serves as a “simple brain”. Intriguingly, gene-gene associations extracted from weight engineering not only recapitulated known gene associations that could explain clinical outcomes of breast cancer patients but importantly capture novel gene associations with immune-related components such as chemokines, carbonic anhydrase, and iron metabolism that modulate immune-related processes and microenvironment in breast cancer microenvironment. This ANN-based algorithm has great promise to extract machine-learned knowledge overlooked by conventional computational methods.

To evaluate the therapeutic effect of immune checkpoint inhibitors (ICIs), Han et al. conducted a pan-cancer analysis which covered 33 different cancers and surveillance of expression signatures for interleukin-17 (IL-17) family genes and their association with tumor prognosis, tumor immune microenvironment composition, and tumor immunotherapy outcomes.

The authors performed a broad array of analyses which include differential expression, gene co-expression, immune subtype, stemness, TME score and immune correlation. Moreover, drug sensitivity analysis inspecting the correlation of IL-17 gene expression with indicators of diagnosis, prognosis, and treatment response of patients were also included. The authors identified distinct expression profiles of IL-17 family members between cancer and normal tissues and further demonstrated that expression of IL-17 family members can serve as signatures for patients’ survival prognosis in some tumors across different immune subtypes. More importantly, they found the expression of IL-17 family genes show a robust correlation with immune cell infiltration, ICI-related immune indicators, and drug sensitivity. This is the first study to systematically investigate the clinical value of IL-17 in cancer diagnosis and treatment outcomes and elucidate the immunotherapeutic role of IL-17 in cancers, which could provide new guidance for cancer treatments.

Adult patients diagnosed with refractory/relapsed (R/R) B-cell acute lymphoblastic leukemia (B-ALL) have poor prognosis. Research by Yang et al. aims to analyze transplantation outcomes after haploidentical hematopoietic stem cell transplantation (haplo-HSCT) in R/R patients with minimal residual disease (MRD) and negative complete remission (MRD-CR) from chimeric antigen receptor (CAR) T cell (CAR-T) therapy. They selected patients who received haplo-HSCT after MRD^-^CR post-chemotherapy over the same period as a control group. This is the first study to compare the clinical prognoses of patients undergoing haplo-HSCT after achieving MRD-CR post-CAR-T therapy and post-chemotherapy. Their long-term follow-up of 31 months found similar survival outcomes and relapsed rates between the chemotherapy and CAR-T groups. Their study also found improved overall survival (OS) and leukemia-free survival (LFS) in R/R patients who received complete remission (CR) from CAR-T than that in relapsed patients after achieving CR2 or later from chemotherapy. In conclusion, this study indicates that by inducing MRD-CR, CAR-T therapy is a safe and feasible bridging regimen for haplo-HSCT without an increased risk of transplanted-related mortality. Patients with a first relapse after conventional chemotherapy could prioritize CAR-T therapy as salvage treatment, and proceed to allo-HSCT following MRD^-^CR.

Overall, the work published in this Research Topic present new insights and will likely have long-lasting effects in the field of cancer immunology and immunotherapy by setting up a roadmap for how systems biology assisted with AI methodologies can bring new insights on disease etiology, dive into cell heterogeneity and inspect disease outcomes. However, the endeavors to gain deeper insights on the interplay between cancer, the TME and the immune system do not end here. For example, biological perspectives or conceptual computational models to elaborate on how cancer and immune cells spatial co-localization and interactions shape phenotypic properties in the TME are needed. This will require the development of innovative spatial systems biology and AI tools to gain mechanistic insights and ultimately reach better immunotherapeutic outcomes in years to come.

## Author contributions

All authors listed have made a substantial, direct, and intellectual contribution to the work and approved it for publication.
